# Supplementary material for: Root mixing effects on belowground decomposition depend on mycorrhizal type
Source: Nat Commun. 2025 Nov 21;16:10274. doi: 10.1038/s41467-025-65163-7 (PMC12639048; doi:10.1038/s41467-025-65163-7)
Supplement: Supplementary file 1 — Supplementary Information [file 41467_2025_65163_MOESM1_ESM.pdf]

Supporting information for

Root mixing effects on belowground decomposition depend on mycorrhizal type

Lei Jiang[1], Stephan Hättenschwiler[2], Ning Ma[3,4], Jiajia Zheng[4,5], Wenhui Shi[1,5], Yeqing Ying[1], Shenggong Li[3,4], Han Yan[6,7] & Liang Kou[4,5,*]

[1]State Key Laboratory for Development and Utilization of Forest Food Resources, Zhejiang A&F University, Hangzhou, 311300, China

[2]CEFE, Univ Montpellier, CNRS, EPHE, IRD, Montpellier, France

[3]National Ecosystem Science Data Center, Key Laboratory of Ecosystem Network Observation and Modeling, Institute of Geographic Sciences and Natural Resources Research, Chinese Academy of Sciences, Beijing 100101, China

[4]College of Resources and Environment, University of Chinese Academy of Sciences, Beijing 100101, China

[5]Qianyanzhou Ecological Research Station, Key Laboratory of Ecosystem Network Observation and Modeling, Institute of Geographic Sciences and Natural Resources Research, Chinese Academy of Sciences, Beijing 100101, China

[6]Freie Universität Berlin, Institut für Biologie, 14195 Berlin, Germany

[7]Berlin-Brandenburg Institute of Advanced Biodiversity Research, 14195 Berlin, Germany

Corresponding author: Liang Kou

Email: koul@igsnrr.ac.cn

Tel: 86-10-6485-7067; Fax: 86-10-6486-8962

Article type: Primary research articles

This file includes:

Supplementary Figures 1-5

Supplementary Tables 1-11

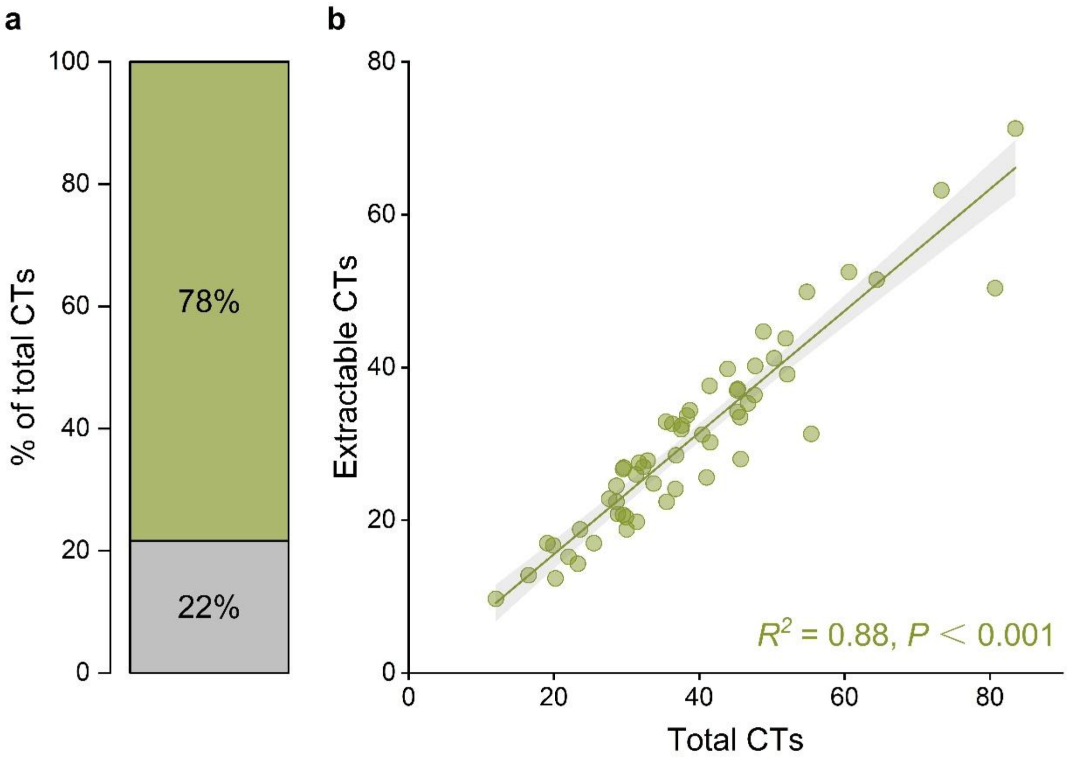

Supplementary Fig. 1 | Relative contribution (%) of extractable condensed tannins (CTs) to total CTs and the relationship between root extractable CTs and total CTs. (a) The green bar shows the proportion of root extractable CTs to total CTs across all litter species (ranging from 12.0 mg g^{-1} to 83.5 mg g^{-1} of total CTs among species, n = 57). **(b)** The positive correlation of total CTs with extractable CTs by fitting linear regressions. Shaded areas show a 95% confidence interval. The statistical significance of the linear regression was assessed using two-sided F-tests. Source data are provided as a Source Data file.

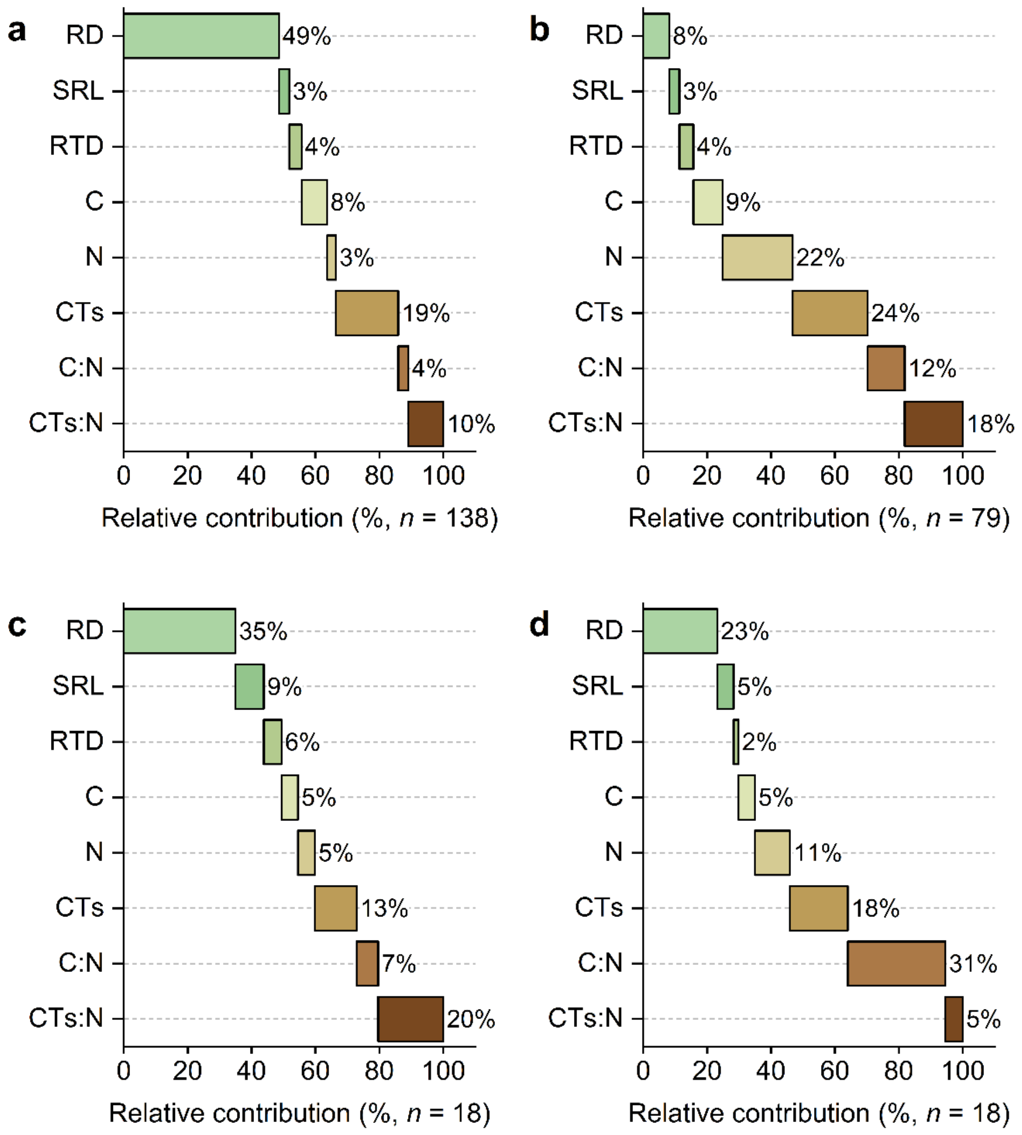

Supplementary Fig. 2 | The relative contribution (%) of eight root traits on mixing effects based on hierarchical partitioning analyses. Specially, community-weighted mean (CWM) trait on mixing effects across (**a**) all data (*n* = 138), across the mixtures with statistically significant **(b)** synergistic effects (*n* = 79) and **(c)** antagonistic effects (*n* = 18); trait dissimilarity (expressed as the absolute difference between the two component species in the mixture) on mixing effects of root combinations with statistically significant **(d)** antagonistic effects (*n* = 18). Source data are provided as a Source Data file.

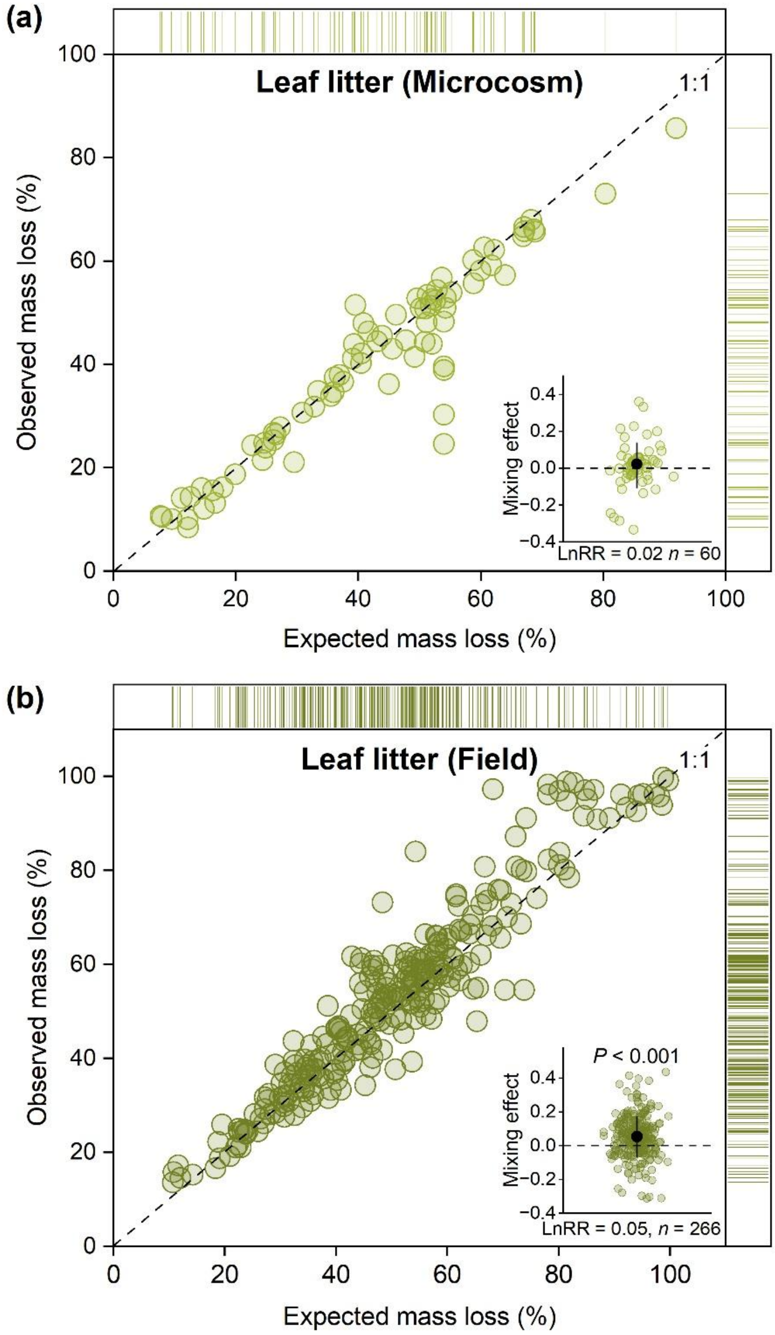

Supplementary Fig. 3 | Observed mass loss as a function of expected mass loss and the corresponding mixing effects for leaf litter decomposition in microcosm studies under controlled conditions (a, *n* = 60) or in field studies (b, *n* = 266). The circles above the 1:1 line indicate that the observed mass loss was greater than the expected mass loss. The insert at the right bottom of each panel shows the corresponding mixing effect (LnRR) of 60 (**a**), and 266 (**b**) paired-species leaf litter combinations, respectively, where the black points and lines represent the means and standard deviations. The significance was based on two-sided *t*-tests. Source data are provided as a Source Data file.

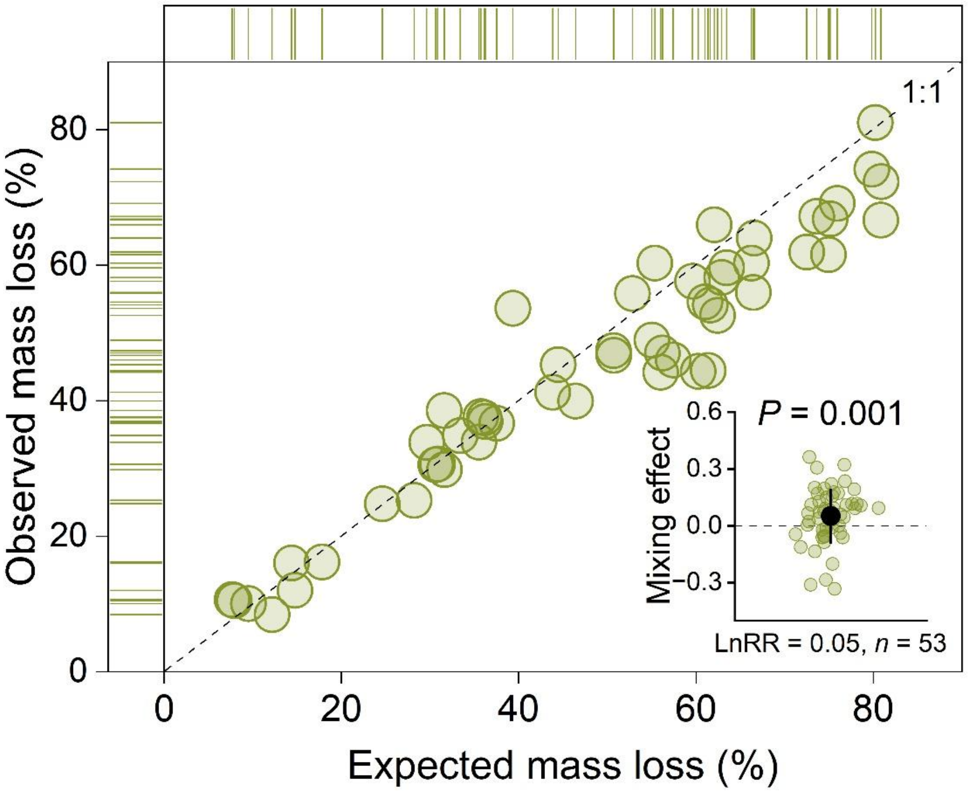

Supplementary Fig. 4 | Observed mass loss as a function of expected mass loss and the corresponding mixing effects for leaf litter decomposition from only the subtropics (*n* = 53). The circles above the 1:1 line indicate that the observed mass loss was greater than the expected mass loss. The insert at the right bottom shows the corresponding mixing effect (LnRR), where the black point and line represent the mean and standard deviation. The significance was based on two-sided *t*-tests. Source data are provided as a Source Data file.

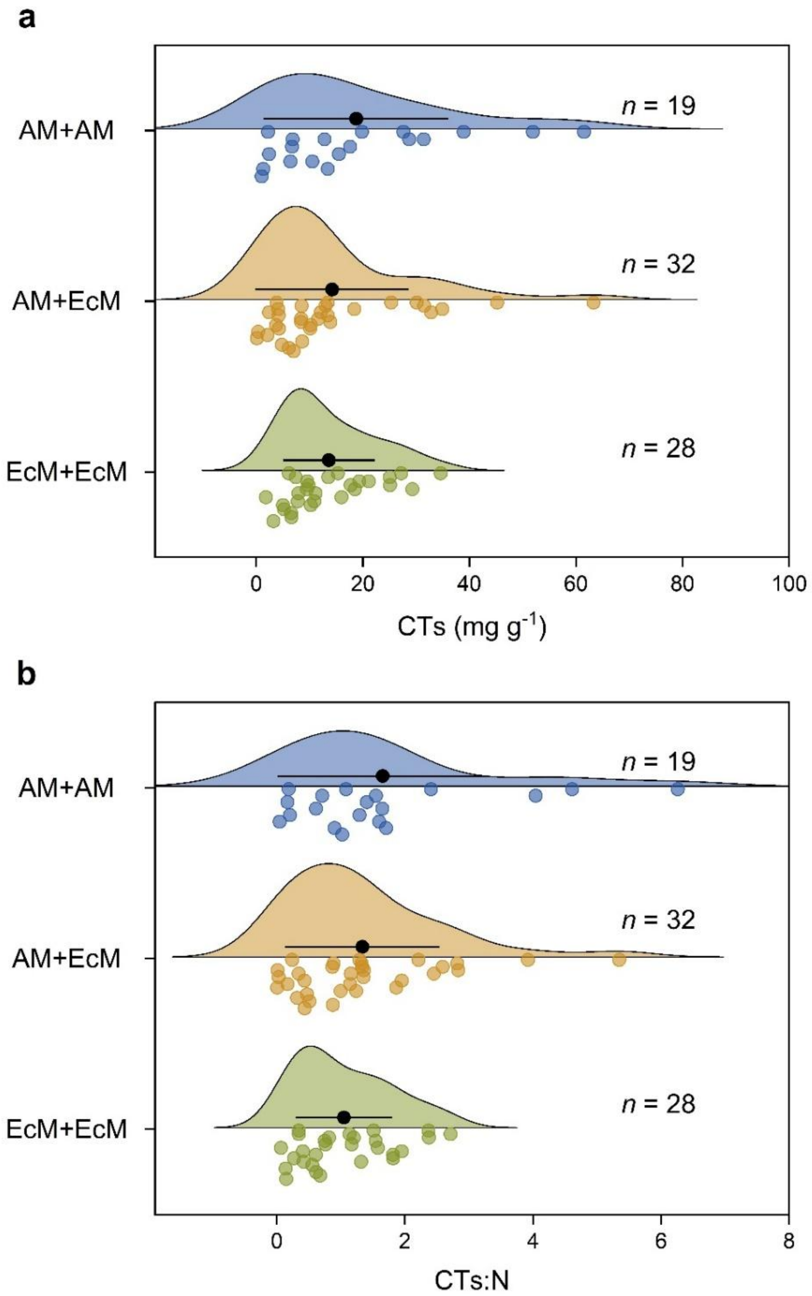

Supplementary Fig. 5 | The difference in root condensed tannins (CTs, a) and root condensed tannins to nitrogen ratio (CTs:N, b) for the three groups of mycorrhizal-type combinations with significant synergistic effects. AM, Arbuscular mycorrhizae; EcM, ectomycorrhizae; blue for AM+AM (*n* = 19), orange for AM+EcM (*n* = 32), green for EcM+EcM (*n* = 28). The black points and lines represent the means and standard deviations. Source data are provided as a Source Data file.

Supplementary Table 1 | List of the 57 studied tree species, their characteristics, and their corresponding absorptive root traits and single species mass loss.

Latin name	Abbr.	Family	Mycorrhizal type	Leaf form	Leaf habit	RD	SRL	RTD	C	N	CTs	C:N	CTs:N	ML
Acer davidii	Acda	Sapindaceae	AM	Broad	Deci	0.35	63.78	0.17	457.70	21.40	50.30	21.39	2.35	13.45
Alniphyllum fortunei	Alfo	Styracaceae	AM	Broad	Deci	0.34	215.78	0.05	429.60	24.50	31.30	17.53	1.28	12.67
Carpinus viminea	Cavi	Betulaceae	EcM	Broad	Deci	0.33	35.80	0.32	425.80	20.40	20.20	20.87	0.99	11.83
Castanea mollissima	Camo	Fagaceae	EcM	Broad	Deci	0.35	80.60	0.14	414.90	13.30	33.70	32.93	2.53	19.20
Castanopsis carlesii	Caca	Fagaceae	EcM	Broad	Ever	0.33	40.74	0.30	507.30	15.40	45.30	32.94	2.94	9.00
Castanopsis eyrei	Caey	Fagaceae	EcM	Broad	Ever	0.33	94.40	0.13	473.20	18.40	43.90	25.72	2.39	10.00
Castanopsis fargesii	Cafa	Fagaceae	EcM	Broad	Ever	0.34	17.13	0.66	418.40	19.20	25.50	21.79	1.33	13.16
Castanopsis fordii	Cafo	Fagaceae	EcM	Broad	Ever	0.32	36.61	0.34	451.80	16.70	35.50	27.05	2.13	9.50
Cerasus serrulata	Cese	Rosaceae	AM	Broad	Deci	0.33	25.04	0.47	436.30	10.80	80.70	40.40	7.47	12.67
Choerospondias axillaris	Chax	Anacardiaceae	AM	Broad	Deci	0.35	92.90	0.12	409.30	17.00	83.50	24.08	4.91	15.30
Cinnamomum camphora	Cica	Lauraceae	AM	Broad	Ever	0.47	25.84	0.23	425.50	24.30	31.70	17.51	1.30	21.48
Cinnamomum micranthum	Cimi	Lauraceae	AM	Broad	Ever	0.44	39.19	0.17	442.10	22.70	30.00	19.48	1.32	18.33
Cinnamomum porrectum	Cipo	Lauraceae	AM	Broad	Ever	0.50	10.84	0.50	479.70	23.80	28.80	20.16	1.21	16.50
Cryptomeria fortunei	Crfo	Cupressaceae	AM	Coni	Ever	0.44	7.78	0.86	481.10	14.90	37.50	32.29	2.52	10.36
Cunninghamia lanceolata	Cula	Cupressaceae	AM	Coni	Deci	0.62	20.21	0.17	481.37	14.10	51.86	34.14	3.68	23.78
Dalbergia hupeana	Dahu	Fabaceae	AM	Broad	Deci	0.32	45.14	0.29	474.30	25.40	38.30	18.67	1.51	20.67
Daphniphyllum oldhami	Daol	Daphniphyllaceae	AM	Broad	Ever	0.34	27.49	0.41	498.90	18.60	41.00	26.82	2.20	14.00
Diospyros morrisiana	Dimo	Ebenaceae	AM	Broad	Ever	0.42	14.10	0.51	466.20	16.40	36.30	28.43	2.21	19.00
Elaeocarpus japonicus	Elja	Elaeocarpaceae	AM	Broad	Ever	0.36	18.89	0.53	431.60	20.60	48.80	20.95	2.37	20.33
Elaeocarpus sylvestris	Elsy	Elaeocarpaceae	AM	Broad	Ever	0.37	31.28	0.31	412.90	11.10	47.60	37.20	4.29	11.50
Euscaphis konishii	Euko	Staphyleaceae	AM	Broad	Ever	0.38	53.91	0.16	436.40	17.90	19.90	24.38	1.11	29.24
Fagus longipetiolata	Falo	Fagaceae	EcM	Broad	Deci	0.34	21.10	0.53	492.60	13.80	38.70	35.70	2.80	9.67
Fokienia hodginsii	Foho	Cupressaceae	AM	Coni	Ever	0.40	12.76	0.62	432.00	14.50	32.90	29.79	2.27	10.00
Garcinia multiflora	Gamu	Clusiaceae	AM	Broad	Ever	0.36	32.88	0.31	443.70	22.70	32.30	19.55	1.42	13.00
Helicteres angustifolia	Hean	Malvaceae	AM	Broad	Deci	0.34	19.42	0.57	464.30	22.40	40.40	20.73	1.80	23.17
Hovenia acerba	Hoac	Rhamnaceae	AM	Broad	Deci	0.38	124.53	0.07	456.30	21.20	23.60	21.52	1.11	23.83
Idesia polycarpa	Idpo	Salicaceae	AM	Broad	Deci	0.35	182.74	0.06	420.20	21.90	35.40	19.19	1.62	24.33
Liquidambar formosana	Lifo	Altingiaceae	AM	Broad	Decis	0.38	56.73	0.17	338.30	12.80	60.60	26.43	4.73	22.61
Liriodendron chinense	Lich	Magnoliaceae	AM	Broad	Deci	0.53	22.56	0.21	465.10	21.20	19.10	21.94	0.90	28.57

Lithocarpus glaber	Ligl	Fagaceae	EcM	Broad	Ever	0.35	116.77	0.13	370.50	13.20	41.50	28.07	3.14	15.90
Litsea elongat	Liel	Lauraceae	AM	Broad	Ever	0.39	23.53	0.36	476.50	24.40	29.50	19.53	1.21	15.33
Machilus pauhoi	Mapa	Lauraceae	AM	Broad	Ever	0.48	87.54	0.06	332.50	15.30	41.40	21.73	2.71	16.78
Machilus phoenicis	Maph	Lauraceae	AM	Broad	Ever	0.50	80.96	0.06	434.30	26.30	23.30	16.51	0.89	14.83
Machilus thunbergii	Matu	Lauraceae	AM	Broad	Ever	0.50	117.41	0.05	434.60	20.80	37.60	20.89	1.81	21.33
Metasequoia glyptostroboides	Megl	Cupressaceae	AM	Coni	Deci	0.41	41.87	0.21	433.40	15.80	45.60	27.43	2.89	14.83
Michelia figo	Mifi	Magnoliaceae	AM	Broad	Ever	0.60	21.44	0.17	460.00	24.10	12.00	19.09	0.50	31.65
Michelia macclurei	Mima	Magnoliaceae	AM	Broad	Ever	0.54	179.82	0.03	355.65	16.77	16.50	21.21	0.98	23.00
Michelia odora	Miod	Magnoliaceae	AM	Broad	Ever	0.38	206.73	0.04	434.20	16.10	31.40	26.97	1.95	12.50
Mytilaria laosensis	Myla	Hamamelidaceae	AM	Broad	Ever	0.38	16.80	0.53	437.10	16.80	45.20	26.02	2.69	12.50
Nyssa sinensis	Nysi	Cornaceae	AM	Broad	Deci	0.36	113.32	0.09	475.70	24.70	28.60	19.26	1.16	15.17
Phoebe bournei	Phbo	Lauraceae	AM	Broad	Ever	0.43	9.60	0.74	477.20	25.10	29.90	19.01	1.19	11.00
Photinia fraseri	Phfr	Rosaceae	AM	Broad	Ever	0.35	31.76	0.33	462.80	9.90	55.40	46.75	5.60	9.17
Pinus massoniana	Pima	Pinaceae	EcM	Coni	Ever	0.40	44.46	0.19	464.07	13.47	45.24	34.46	3.36	14.41
Quercus acutissima	Quac	Fagaceae	EcM	Broad	Deci	0.34	119.09	0.11	434.90	14.80	54.80	29.39	3.70	15.30
Quercus fabri	Qufa	Fagaceae	EcM	Broad	Deci	0.35	95.83	0.13	393.40	12.60	27.60	29.58	2.19	17.38
Sapium discolor	Sadi	Euphorbiaceae	AM	Broad	Deci	0.37	16.97	0.55	447.40	20.80	52.10	21.51	2.50	20.50
Schima argentea	Scar	Theaceae	AM	Broad	Ever	0.34	17.24	0.63	497.30	18.50	64.40	26.88	3.48	13.17
Schima superba	Scsu	Theaceae	AM	Broad	Ever	0.35	112.05	0.09	426.00	14.80	73.30	28.78	4.95	15.29
Semiliquidambar cathayensis	Seca	Altingiaceae	AM	Broad	Ever	0.36	62.71	0.16	415.00	11.30	45.70	36.73	4.04	14.33
Sloanea sinensis	Slsi	Elaeocarpaceae	AM	Broad	Ever	0.36	86.61	0.12	400.00	12.30	28.60	32.52	2.33	13.33
Styrax suberifolius	Stsu	Styracaceae	AM	Broad	Ever	0.33	66.67	0.19	459.80	20.50	36.70	22.43	1.79	9.33
Taxus wallichiana	Tawa	Taxaceae	AM	Coni	Ever	0.50	136.05	0.04	397.80	19.80	29.50	20.09	1.49	14.61
Toona ciliata	Toci	Meliaceae	AM	Broad	Deci	0.42	106.69	0.07	468.40	21.30	46.70	21.99	2.19	11.94
Toona sinensis	Tosi.	Meliaceae	AM	Broad	Ever	0.49	25.18	0.22	446.30	23.40	47.70	19.07	2.04	17.75
Toxicodendron succedaneum	Tosu	Anacardiaceae	AM	Broad	Deci	0.36	189.55	0.05	430.20	21.70	36.80	19.82	1.70	19.50
Vernicia montana	Vemo	Euphorbiaceae	AM	Broad	Deci	0.40	21.02	0.38	425.30	25.30	22.00	16.81	0.87	17.50
Zanthoxylum ailanthoides	Zaai	Rutaceae	AM	Broad	Deci	0.32	72.81	0.17	482.00	22.40	29.60	21.52	1.32	30.71

Note: Mycorrhizal types include arbuscular mycorrhizal (AM) and ectomycorrhizal (EcM) species; Leaf forms include broadleaf (Broad) and coniferous (Coni); Leaf habits include evergreen (Ever) and deciduous (Deci); RD, root diameter (mm); SRL, specific root length (m g^{-1}); RTD, root tissue density (g cm^{-3}); C, root carbon concentration (mg g^{-1}); N, root nitrogen concentration (mg g^{-1}); CTs, root total condensed tannins concentration (mg g^{-1}); C:N, root carbon to nitrogen ratio; CTs:N, root condensed tannins to nitrogen ratio. Ratios are based on mass. ML, mass loss (%) after 12 weeks of incubation.

Supplementary Table 2 | Mean, range and variation of absorptive root traits across the 57 studied tree species.

Trait	Mean	SD	Min	Max	CV (%)
RD (mm)	0.39	0.07	0.32	0.62	18.3
SRL (m g^{-1})	64.75	54.43	7.78	215.78	84.1
RTD (g cm^{-3})	0.27	0.21	0.03	0.86	77.1
C (mg g^{-1})	441.04	37.08	332.50	507.30	8.4
N (mg g^{-1})	18.59	4.51	9.90	26.30	24.3
CTs (mg g^{-1})	39.01	14.67	12.00	83.50	37.6
C:N	25.15	6.61	16.51	46.75	26.3
CTs:N	2.34	1.35	0.50	7.47	57.7
ML (%)	16.60	5.64	9.00	31.65	34.0

SD, standard deviation (n = 57); Min, minimum value; Max, maximum value; CV, coefficient of variation; RD, root diameter; SRL, specific root length; RTD, root tissue density; C, root carbon concentration; N, root nitrogen concentration; CTs, root condensed tannins concentration; C:N, root carbon to nitrogen ratio; CTs:N, root condensed tannins to nitrogen ratio. Ratios are based on mass. ML, mass loss.

Supplementary Table 3 | Mean, range, and variation of community-weighted mean (CWM) values of absorptive root traits for 138 paired-species combinations, and trait dissimilarity (defined as the absolute difference between the two species in the mixture) for the same 138 paired-species combinations or for only the 79 paired-species combinations with statistically significant synergistic effects.

Trait	CWM (*n* = 138)					Absolute difference (*n* = 138)					Absolute difference (*n* = 79)				
	Mean	SD	Min	Max	CV (%)	Mean	SD	Min	Max	CV (%)	Mean	SD	Min	Max	CV (%)
RD	0.38	0.05	0.32	0.57	13.6	0.06	0.07	0.00	0.30	108.8	0.05	0.06	0.00	0.21	109.3
SRL	65.60	35.80	11.85	199.26	54.6	53.11	42.17	0.81	176.59	79.4	57.14	43.32	0.81	175.59	75.8
RTD	0.26	0.13	0.05	0.66	52.0	0.20	0.18	0.00	0.72	88.0	0.21	0.17	0.00	0.71	81.9
C	439.43	24.94	375.60	499.95	5.7	41.74	30.86	0.60	143.20	73.9	46.80	33.03	0.60	143.20	70.6
N	17.45	3.01	11.55	25.30	17.2	4.94	3.31	0.03	13.10	67.1	4.96	3.38	0.03	13.00	68.1
CTs	38.34	9.05	15.55	67.80	23.6	14.09	11.96	0.06	63.30	84.9	15.11	13.48	0.10	63.30	89.2
C:N	26.54	4.49	17.01	37.41	16.9	6.83	4.82	0.05	25.53	70.5	7.11	5.12	0.05	25.53	72.0
CTs:N	2.39	0.80	0.70	5.18	33.4	1.18	1.10	0.00	6.26	93.2	1.31	1.20	0.01	6.26	91.8
ML_{obs}	19.17	4.95	10.28	33.05	25.8	19.17	4.95	10.28	33.05	25.8	20.77	4.27	10.84	30.18	20.5
LnRR	0.17	0.24	-0.50	0.68	147.7	0.17	0.24	-0.50	0.68	147.7	0.33	0.15	0.09	0.68	46.7

SD, standard deviation; Min, minimum value; Max, maximum value; CV, coefficient of variation; RD, root diameter (mm); SRL, specific root length (m g^{-1}); RTD, root tissue density (g cm^{-3}); C, root carbon concentration (mg g^{-1}); N, root nitrogen concentration (mg g^{-1}); CTs, total condensed tannins concentration (mg g^{-1}); C:N, root carbon to nitrogen ratio; CTs:N, root condensed tannins to nitrogen ratio; Ratios are based on mass; ML_{obs}, observed mass loss (%) of 138 paired-species combinations; LnRR, mixing effect of 138 paired-species combinations or 79 synergistic-effect combinations.

Supplementary Table 4 | Relationships between community-weighted mean (CWM) traits or trait dissimilarity (absolute difference) of absorptive root traits and mixing effects on root decomposition for all combinations (*n* = 138), the combinations with synergistic effects (*n* = 79), and the combinations with antagonistic effects (*n* = 18).

Trait	All combinations (*n* = 138)								Synergistic-effect combinations (*n* = 79)								Antagonistic-effect combinations (*n* = 18)							
	CWM				Absolute difference				CWM				Absolute difference				CWM				Absolute difference			
	F	*df*	*r*	*P*	*F*	*df*	*r*	*P*	*F*	*df*	*r*	*P*	*F*	*df*	*r*	*P*	*F*	*df*	*r*	*P*	*F*	*df*	*r*	*P*
RD	8.75	1	-0.25	**0.004**	4.63	1	-0.18	**0.033**	0.14	1	-0.04	0.707	0.01	1	0.01	0.931	5.16	1	-0.49	**0.037**	3.60	1	-0.43	0.076
SRL	0.02	1	-0.01	0.898	0.16	1	0.03	0.688	1.42	1	-0.13	0.238	0.01	1	-0.01	0.904	2.24	1	0.35	0.154	1.30	1	0.27	0.272
RTD	0.71	1	0.07	0.400	0.06	1	-0.02	0.803	1.85	1	0.15	0.178	0.17	1	0.05	0.677	0.27	1	-0.13	0.614	0.05	1	-0.06	0.821
C	1.84	1	-0.12	0.177	3.89	1	0.17	0.051	1.48	1	0.14	0.228	0.00	1	0.00	0.995	0.07	1	0.06	0.799	0.57	1	-0.18	0.463
N	0.14	1	-0.03	0.713	1.42	1	0.10	0.236	3.79	1	0.22	0.055	1.95	1	0.16	0.167	0.00	1	0.02	0.945	0.25	1	0.12	0.623
CTs	1.85	1	-0.12	0.177	9.95	1	0.26	**0.002**	1.49	1	-0.14	0.225	7.72	1	0.30	**0.007**	1.69	1	0.10	0.213	3.19	1	-0.41	0.093
C:N	0.00	1	0.00	0.972	2.95	1	0.15	0.088	1.23	1	-0.13	0.272	5.00	1	0.25	**0.028**	0.01	1	-0.31	0.920	2.84	1	-0.39	0.111
CTs:N	0.17	1	-0.04	0.682	10.08	1	0.26	**0.002**	1.33	1	-0.13	0.253	6.69	1	0.28	**0.012**	1.38	1	-0.28	0.257	0.22	1	-0.12	0.644

The statistical significance of the linear regression was assessed using two-sided *F*-tests. Significant values are in bold. RD, root diameter (mm); SRL, specific root length (m g^{-1}); RTD, root tissue density (g cm^{-3}); C, root carbon concentration (mg g^{-1}); N, root nitrogen concentration (mg g^{-1}); CTs, total condensed tannins concentration (mg g^{-1}); C:N, root carbon to nitrogen ratio; CTs:N, root condensed tannins to nitrogen ratio. Ratios are based on mass. Mixing effects of paired-species were calculated as the response ratio of the differences between observed and expected mass loss, LnRR = $\ln(ML_{obs}/ML_{exp})$. Source data are provided as a Source Data file.

Supplementary Table 5 | Mean, range, and variation of absorptive root traits for arbuscular mycorrhizal (AM) and ectomycorrhizal (EcM) species.

Trait	AM (*n* = 46)					EcM (*n* = 11)				
	Mean	SD	Min	Max	CV (%)	Mean	SD	Min	Max	CV (%)
RD	0.41	0.01	0.32	0.62	18.38	0.34	0.01	0.32	0.40	6.10
SRL	64.96	8.55	7.78	215.78	89.27	63.87	11.50	17.13	119.09	59.70
RTD	0.27	0.03	0.03	0.86	79.95	0.27	0.06	0.11	0.66	67.81
C	441.14	5.36	332.50	498.90	8.25	440.62	12.59	370.50	507.30	9.48
N	19.13	0.67	9.90	26.30	23.69	15.57	0.82	12.60	20.40	17.42
CTs	39.38	2.30	12.00	83.50	39.68	37.45	3.07	20.20	54.80	27.21
C:N	24.24	0.99	16.51	46.75	27.59	28.95	1.48	20.87	35.70	16.90
CTs:N	2.30	0.21	0.50	7.47	63.12	2.50	0.25	0.99	3.70	32.95
ML	17.41	0.85	9.17	31.65	33.19	13.21	1.06	9.00	19.20	26.49

SD, standard deviation; Min, minimum value; Max, maximum value; CV, coefficient of variation; RD, root diameter (mm); SRL, specific root length ($m\ g^{-1}$); RTD, root tissue density ($g\ cm^{-3}$); C, root carbon concentration ($mg\ g^{-1}$); N, root nitrogen concentration ($mg\ g^{-1}$); CTs, total condensed tannins concentration ($mg\ g^{-1}$); C:N, root carbon to nitrogen ratio; CTs:N, root condensed tannins to nitrogen ratio; Ratios are based on mass; ML, mass loss (%) of 57 tree species.

Supplementary Table 6 | Results of different iteration numbers and sensitivity analysis for absorptive root traits and mass loss to test for differences between arbuscular mycorrhizal (AM) and ectomycorrhizal (EcM) species.

Trait	500		1000		2000		5000		Relative variation (%)
	Significant numbers	*P*-value	Significant numbers	*P*-value	Significant numbers	*P*-value	Significant numbers	*P*-value	
RD	411	**0.033**	822	**0.032**	1637	**0.034**	4122	**0.034**	5.91
SRL	29	0.489	63	0.498	111	0.508	318	0.501	3.92
RTD	23	0.493	48	0.504	115	0.501	267	0.500	2.12
C	21	0.508	54	0.485	111	0.485	305	0.486	4.92
N	237	0.152	523	0.138	1004	0.136	2466	0.148	11.41
CTs	33	0.465	74	0.449	112	0.470	299	0.461	4.64
C:N	251	0.169	485	0.178	964	0.166	2366	0.174	7.21
CTs:N	70	0.468	128	0.457	235	0.457	569	0.457	2.42
ML	280	0.111	469	0.128	1079	0.114	2538	0.124	5.25

The significance was reported for each trait tested using two sided *t*-tests. Significant values are in bold. RD, root diameter (mm); SRL, specific root length (m g^{-1}); RTD, root tissue density (g cm^{-3}); C, root carbon concentration (mg g^{-1}); N, root nitrogen concentration (mg g^{-1}); CTs, total condensed tannins concentration (mg g^{-1}); C:N, root carbon to nitrogen ratio; CTs:N, root condensed tannins to nitrogen ratio; Ratios are based on mass; ML, mass loss (%) of 57 tree species. Source data are provided as a Source Data file.

Supplementary Table 7 | Results of different iteration numbers and sensitivity analysis to test for differences in mixing effects among the three mycorrhizal-type groups for either all mixtures (upper line with *n* = 138) or only the mixtures showing significant synergistic effects (lower line with *n* = 79).

		500		1000		2000		5000		Relative variation (%)
		Significant numbers	*P*-value	Significant numbers	*P*-value	Significant numbers	*P*-value	Significant numbers	*P*-value	
LnRR	138	426	**0.035**	845	**0.035**	1674	**0.036**	4249	**0.034**	6.17
	79	118	0.260	284	0.259	504	0.264	1256	0.269	3.82

The significance was reported for mixing effects tested using two sided *t*-tests. Significant values are in bold. LnRR, mixing effects; 138, all combinations = 54 combinations (pure AM mixtures) +47 combinations (mixed AM/EcM mixtures) +37 combinations (pure EcM mixtures); 79, statistically significant combiantions = 19 combinations (pure AM mixtures) +32 combinations (mixed AM/EcM mixtures) + 28 combinations (pure EcM mixtures); AM, arbuscular mycorrhizae; EcM, ectomycorrhizae. Source data are provided as a Source Data file.

Supplementary Table 8 | The relative contribution (%) of trait dissimilarity in root mixtures composed of exclusively arbuscular mycorrhizal (AM) roots across all AM + AM mixtures (*n* = 54), for the mixtures with synergistic effects (*n* = 19), and for the mixtures with antagonistic effects (*n* = 12) based on hierarchical partitioning analyses, and the results from linear regressions testing how mixing effect on root decomposition depend on trait dissimilarity.

Trait	All combinations (*n* = 54)					Synergistic-effect combinations (*n* = 19)					Antagonistic-effect combinations (*n* = 12)				
	Relative contribution (%)	*F*	*df*	*r*	*P*	Relative contribution (%)	*F*	*df*	*r*	*P*	Relative contribution (%)	*F*	*df*	*r*	*P*
RD	10.4	0.27	1	-0.07	0.604	22.1	2.29	1	0.34	0.148	5.4	1.30	1	-0.34	0.281
SRL	8.2	1.51	1	0.17	0.225	16.2	2.03	1	0.33	0.173	12.5	2.39	1	0.44	0.153
RTD	1.4	0.47	1	0.09	0.497	3.6	0.08	1	0.07	0.775	2.2	0.00	1	-0.01	0.979
C	16.5	2.94	1	0.23	0.092	13.5	0.09	1	-0.07	0.773	33.7	7.52	1	-0.66	**0.021**
N	5.9	1.04	1	0.14	0.313	3.9	0.00	1	0.01	0.958	11.1	0.56	1	0.23	0.473
CTs	24.4	6.31	1	0.33	**0.015**	21.4	3.89	1	0.43	0.065	8.0	1.75	1	-0.39	0.216
C:N	7.9	0.18	1	0.06	0.671	8.9	1.36	1	0.27	0.260	19.1	2.00	1	-0.41	0.187
CTs:N	25.2	5.36	1	0.31	**0.025**	10.3	1.78	1	0.31	0.199	8.0	1.35	1	-0.35	0.272

The statistical significance of the linear regression was assessed using two-sided *F*-tests. Significant values are in bold. RD, root diameter (mm); SRL, specific root length (m g^{-1}); RTD, root tissue density (g cm^{-3}); C, root carbon concentration (mg g^{-1}); N, root nitrogen concentration (mg g^{-1}); CTs, total condensed tannins concentration (mg g^{-1}); C:N, root carbon to nitrogen ratio; CTs:N, root condensed tannins to nitrogen ratio. Ratios are based on mass. Source data are provided as a Source Data file.

Supplementary Table 9 | The relative contribution (%) of trait dissimilarity in root mixtures composed of roots from one arbuscular mycorrhizal species (AM) and one ectomycorrhizal species (EcM) across all mixtures (*n* = 47), for the mixtures with synergistic effects (*n* = 32), and for the mixtures with antagonistic effects (*n* = 4) based on hierarchical partitioning analyses, and the results from linear regressions testing how mixing effect on root decomposition depend on trait dissimilarity.

Trait	All combinations (*n* = 47)					Synergistic-effect combinations (*n* = 32)					Antagonistic-effect combinations (*n* = 4)				
	Relative contribution (%)	*F*	*df*	*r*	*P*	Relative contribution (%)	*F*	*df*	*r*	*P*	Relative contribution (%)	*F*	*df*	*r*	*P*
RD	3.8	0.42	1	-0.10	0.519	27.0	1.73	1	-0.23	0.198	10.2	0.88	1	-0.55	0.447
SRL	1.1	0.09	1	-0.04	0.770	7.8	0.59	1	-0.14	0.447	11.2	0.45	1	-0.43	0.570
RTD	6.7	0.73	1	-0.13	0.396	3.7	0.03	1	0.03	0.869	10.5	1.26	1	-0.62	0.378
C	18.7	0.41	1	0.09	0.526	1.8	0.10	1	-0.06	0.749	10.4	1.07	1	-0.59	0.409
N	2.7	0.54	1	0.11	0.466	0.7	0.01	1	-0.02	0.914	17.4	6.90	1	0.88	0.120
CTs	25.9	3.55	1	0.27	0.066	28.2	2.77	1	0.29	0.107	10.7	2.09	1	0.72	0.285
C:N	8.5	1.61	1	0.19	0.212	2.2	0.19	1	0.08	0.662	16.6	3.88	1	0.81	0.188
CTs:N	32.7	4.90	1	0.31	**0.032**	28.7	2.82	1	0.29	0.103	13.1	3.22	1	0.79	0.215

The statistical significance of the linear regression was assessed using two-sided *F*-tests. Significant values are in bold. RD, root diameter (mm); SRL, specific root length (m g^{-1}); RTD, root tissue density (g cm^{-3}); C, root carbon concentration (mg g^{-1}); N, root nitrogen concentration (mg g^{-1}); CTs, total condensed tannins concentration (mg g^{-1}); C:N, root carbon to nitrogen ratio; CTs:N, root condensed tannins to nitrogen ratio. Ratios are based on mass. Source data are provided as a Source Data file.

Supplementary Table 10 | The relative contribution (%) of trait dissimilarity in root mixtures composed of exclusively ectomycorrhizal (EcM) roots across all EcM + EcM mixtures (*n* = 37), and for the mixtures with synergistic effects (*n* = 28) based on hierarchical partitioning analyses, and the results from linear regressions testing how mixing effect on root decomposition depend on trait dissimilarity.

Trait	All combinations (*n* = 37)					Synergistic-effect combinations (*n* = 28)				
	Relative contribution (%)	*F*	*df*	*r*	*P*	Relative contribution (%)	*F*	*df*	*r*	*P*
RD	10.5	2.10	1	-0.24	0.156	0.6	0.15	1	0.08	0.697
SRL	2.0	0.06	1	-0.04	0.804	9.0	1.48	1	-0.23	0.235
RTD	1.2	0.27	1	0.09	0.609	1.6	0.14	1	-0.07	0.715
C	2.3	0.73	1	-0.14	0.399	5.4	0.33	1	0.11	0.568
N	21.5	10.08	1	0.47	**0.003**	52.5	17.89	1	0.64	**<0.001**
CTs	6.7	3.29	1	0.29	0.078	3.0	1.18	1	0.21	0.288
C:N	48.9	19.51	1	0.60	**<.001**	22.1	10.84	1	0.54	**0.003**
CTs:N	6.9	4.24	1	0.33	**0.047**	5.8	2.44	1	0.29	0.130

The statistical significance of the linear regression was assessed using two-sided *F*-tests. Significant values are in bold. RD, root diameter (mm); SRL, specific root length (m g^{-1}); RTD, root tissue density (g cm^{-3}); C, root carbon concentration (mg g^{-1}); N, root nitrogen concentration (mg g^{-1}); CTs, total condensed tannins concentration (mg g^{-1}); C:N, root carbon to nitrogen ratio; CTs:N, root condensed tannins to nitrogen ratio. Ratios are based on mass. Source data are provided as a Source Data file.

Supplementary Table 11 | Properties of homogenized soils used for the microcosm decomposition experiment of absorptive roots.

Soil properties	Value (mean ± standard deviation, *n* = 4)
pH	4.33 ± 0.02
NH_4^+-N (mg kg^{-1})	42.52 ± 2.24
NO_3-N (mg kg^{-1})	5.70 ± 0.40
Available phosphorus (AP, mg kg^{-1})	5.23 ± 0.12
Total carbon (TC, mg g^{-1})	35.90 ± 0.31
Total nitrogen (TN, mg g^{-1})	2.17 ± 0.03
Total phosphorus (TP, mg g^{-1})	0.18 ± 0.01
TC:TN	16.72 ± 0.23
TN:TP	12.28 ± 0.39
