# Supplementary material for: Root mixing effects on belowground decomposition depend on mycorrhizal type
Source: Nat Commun. 2025 Nov 21;16:10274. doi: 10.1038/s41467-025-65163-7 (PMC12639048; doi:10.1038/s41467-025-65163-7)
Supplement: Supplementary file 2 — Reporting Summary [file 41467_2025_65163_MOESM2_ESM.pdf]

nature portfolio

Corresponding author(s): Liang Kou

Last updated by author(s): Oct 7, 2025

Reporting Summary

Nature Portfolio wishes to improve the reproducibility of the work that we publish. This form provides structure for consistency and transparency in reporting. For further information on Nature Portfolio policies, see our Editorial Policies and the Editorial Policy Checklist.

Statistics

For all statistical analyses, confirm that the following items are present in the figure legend, table legend, main text, or Methods section.

n/a	Confirmed	
☐	☒	The exact sample size (*n*) for each experimental group/condition, given as a discrete number and unit of measurement
☐	☒	A statement on whether measurements were taken from distinct samples or whether the same sample was measured repeatedly
☐	☒	The statistical test(s) used AND whether they are one- or two-sided<br>*Only common tests should be described solely by name; describe more complex techniques in the Methods section.*
☐	☒	A description of all covariates tested
☐	☒	A description of any assumptions or corrections, such as tests of normality and adjustment for multiple comparisons
☐	☒	A full description of the statistical parameters including central tendency (e.g. means) or other basic estimates (e.g. regression coefficient) AND variation (e.g. standard deviation) or associated estimates of uncertainty (e.g. confidence intervals)
☐	☒	For null hypothesis testing, the test statistic (e.g. *F*, *t*, *r*) with confidence intervals, effect sizes, degrees of freedom and *P* value noted<br>*Give P values as exact values whenever suitable.*
☒	☐	For Bayesian analysis, information on the choice of priors and Markov chain Monte Carlo settings
☒	☐	For hierarchical and complex designs, identification of the appropriate level for tests and full reporting of outcomes
☒	☐	Estimates of effect sizes (e.g. Cohen's *d*, Pearson's *r*), indicating how they were calculated

Our web collection on statistics for biologists contains articles on many of the points above.

Software and code

Policy information about availability of computer code

Data collection	Root samples were dissected and scanned using an Epson Expression 10000 XL scanner (Seiko Epson Corporation, Suwa, Nagano, Japan).<br>Root traits were measured using WinRHIZO Arabidopsis version 2012b (Regents Instruments Inc., Quebec, Canada).<br>Root C and N concentrations were measured using a Vario Macro cube elemental analyser (Elementar Analysensysteme GmbH, Langenselbold, Germany).
Data analysis	Data analyses were performed using the R version 4.3.2 (https://www.R-project.org/) and all figures were plotted in OriginPro (2024, Origin Lab., Hampton, MA, USA). Details were reported in the Methods section.

For manuscripts utilizing custom algorithms or software that are central to the research but not yet described in published literature, software must be made available to editors and reviewers. We strongly encourage code deposition in a community repository (e.g. GitHub). See the Nature Portfolio guidelines for submitting code & software for further information.

Data

Policy information about availability of data

All manuscripts must include a data availability statement. This statement should provide the following information, where applicable:

- Accession codes, unique identifiers, or web links for publicly available datasets
- A description of any restrictions on data availability
- For clinical datasets or third party data, please ensure that the statement adheres to our policy

Data and R code in the support of these findings have been deposited in the Figshare repository: https://doi.org/10.6084/m9.figshare.29151101.

Research involving human participants, their data, or biological material

Policy information about studies with human participants or human data. See also policy information about sex, gender (identity/presentation), and sexual orientation and race, ethnicity and racism.

Reporting on sex and gender	N/A
Reporting on race, ethnicity, or other socially relevant groupings	N/A
Population characteristics	N/A
Recruitment	N/A
Ethics oversight	N/A

Note that full information on the approval of the study protocol must also be provided in the manuscript.

Field-specific reporting

Please select the one below that is the best fit for your research. If you are not sure, read the appropriate sections before making your selection.

☐ Life sciences ☐ Behavioural & social sciences ☒ Ecological, evolutionary & environmental sciences

For a reference copy of the document with all sections, see nature.com/documents/nr-reporting-summary-flat.pdf

Ecological, evolutionary & environmental sciences study design

All studies must disclose on these points even when the disclosure is negative.

Study description	We sampled absorptive roots of 57 tree species and obtained 138 two-species mixtures at two sites of subtropical forests in China. We performed a microcosm experiment to quantify the decomposition rate of absorptive roots for these litter treatments. To reveal trait controls over belowground diversity effects on root decomposition, we further integrated root morphological and chemical traits.
Research sample	In total, 585 microcosms ((57 tree species + 138 two-species mixture) × 3 replicates) were set up in this study.
Sampling strategy	For each species, roots were collected in the field from three to five healthy and mature trees with a similar diameter at breast height and with a distance of at least 10 m between individual trees. We determined four 1 m × 1 m plots, one in each cardinal direction, around each target individual tree at the same distance for an individual tree, but ranging between 1.5 m and 2.5 m from the tree stem among individual trees. In each plot, we excavated roots from the surface soil (0-20 cm) and traced them back to the stem to verify their identity. Then, the intact fine roots including at least the first five root orders were carefully cut from the main lateral roots. All root samples were sealed into valve bags and transported to the laboratory typically within two hours. We collected the surface soil (0-20 cm) within the root sampling plots for all individuals of the 57 tree species to represent the sampling location from the two forest sites.
Data collection	Data collection was performed by Lei Jiang, Ning Ma, Han Yan, and Jiajia Zheng during the field and laboratory experiment. Briefly, Lei Jiang, Jiajia Zheng and Han Yan used instruments for root trait measurements, and Ning Ma used paper and computer for recording. Lei Jiang set up the indoor microcosm experiment with the help of Jiajia Zheng, and Han Yan and Ning Ma weighed and recorded them on a balance.
Timing and spatial scale	We conducted field sampling in the autumn of 2018 and 2020. We conducted our study across two sites, including (i) Qianyanzhou Ecological Station (26°44′39″N, 115°03′33″E) and (ii) Yangming Mountain (25°39′N, 114°18′E) in Jiangxi Province.
Data exclusions	Once we compiled the dataset according to our criteria, we did not deliberately delete any data.
Reproducibility	We did not repeat the experiment. But we have replicates (see 'Sampling strategy' above).
Randomization	For each species, three to five healthy and mature trees with a similar diameter at breast height were randomly selected with a

spacing of at least 10 m apart to ensure independence. In the root decomposition experiment, each litter bag was randomly embedded in the corresponding microcosm.

Blinding: Blinding was not applicable since this is a field investigation experiment.

Did the study involve field work? [x] Yes [ ] No

Field work, collection and transport

Field conditions	The study was conducted in subtropical (two sites) forests in China. Both sites are characterized by similar climatic conditions, typical for the subtropical climate zone, with mean annual temperatures and precipitations of 17.9 °C and 1475 mm at Qianyanzhou Ecological Station site, and 17.7 °C and 1587 mm at Yangming Mountain site.
Location	These two sites were located at (i) Qianyanzhou Ecological Station (26°44′39″N, 115°03′33″E) in Jiangxi Province; and (ii) Yangming Mountain (25°39′N, 114°18′E) in Jiangxi Province.
Access & import/export	We needed no other permits for this study, and all site managers provided their full permission for us to access their land for fieldwork.
Disturbance	Disturbance was limited by backfilling topsoil after the sampling was concluded.

Reporting for specific materials, systems and methods

We require information from authors about some types of materials, experimental systems and methods used in many studies. Here, indicate whether each material, system or method listed is relevant to your study. If you are not sure if a list item applies to your research, read the appropriate section before selecting a response.

Materials & experimental systems

n/a	Involved in the study
[x]	[ ] Antibodies
[x]	[ ] Eukaryotic cell lines
[x]	[ ] Palaeontology and archaeology
[x]	[ ] Animals and other organisms
[x]	[ ] Clinical data
[x]	[ ] Dual use research of concern
[x]	[ ] Plants

Methods

n/a	Involved in the study
[x]	[ ] ChIP-seq
[x]	[ ] Flow cytometry
[x]	[ ] MRI-based neuroimaging

Plants

Seed stocks	For each species, three to five healthy and mature trees with a similar diameter at breast height were randomly selected, with a spacing of at least 10 m apart to ensure independence, and the intact fine roots were excavated by exposing the main lateral roots, traced back to individual trees and cleaned .
Novel plant genotypes	N/A
Authentication	N/A
